# Joint associations between exercising and classical cardiometabolic risk factors with all-cause, premature and cardiovascular mortality: a prospective cohort study among Mexican adults

**DOI:** 10.3389/fendo.2026.1850784

**Published:** 2026-07-27

**Authors:** Raquel Pastor-Cisneros, Duarte Henriques-Neto, Damián Pereira-Payo, Adilson Marques, Gerson Ferrari, Alex Barreto de Lima, Miguel Peralta

**Affiliations:** 1 Physical Activity for Education, Performance and Health (PAEPH) Research Group, Faculty of Sport Sciences, University of Extremadura, Cáceres, Spain; 2 Research Center in Sports Sciences, Health Sciences and Human Development (CIDESD), Maia, Portugal; 3 University of Maia, Maia, Portugal; 4 Health, Economy, Motricity and Education (HEME) Research Group, Faculty of Sport Sciences, University of Extremadura, Cáceres, Spain; 5 CIPER, Faculdade de Motricidade Humana, Universidade de Lisboa, Cruz Quebrada, Portugal; 6 Universidad de Santiago de Chile (USACH), Escuela de Ciencias de la Actividad Física, el Deporte y la Salud, Santiago, Chile; 7 Facultad de Ciencias de la Salud, Universidad Autónoma de Chile, Santiago, Chile; 8 Course of Physical Education, University of Nilton Lins, Manaus, Brazil; 9 ISAMB, Faculdade de Medicina, Universidade de Lisboa, Lisboa, Portugal

**Keywords:** death, diabetes, hypertension, obesity, physical activity, prevention

## Abstract

**Background:**

Cardiovascular diseases are the leading cause of mortality worldwide, with a particularly high burden in low- and middle-income countries such as Mexico. Cardiometabolic risk factors are strongly associated with increased mortality. Although exercise is well established as protective against mortality, evidence on its joint associations with these risk factors in high-risk populations remains limited. This study aimed to examine the joint associations of exercise and classical cardiometabolic risk factors with all-cause, premature, and cardiovascular mortality among Mexican adults.

**Methods:**

This prospective cohort study analyzed data from 155156 adults (mean age, 52.3 years; 67.1% women) from the Mexico City Prospective Study. Exercise behavior and cardiometabolic risk factors (hypertension, diabetes, obesity, and smoking) were assessed at baseline. Mortality outcomes (all-cause, premature <75 years, and cardiovascular mortality) were tracked over a median follow-up of 18.26 years. Cox proportional hazards models, adjusted for sociodemographic characteristics and additional risk factors, were used to estimate associations. Joint exposure categories combining exercise with each cardiometabolic risk factor were analyzed.

**Results:**

During follow-up, 27510 deaths were recorded, including 13970 premature and 9192 cardiovascular deaths. Individuals who exercised had significantly lower mortality risk compared to non-exercisers. Exercise was associated with a 12-16% reduction in all-cause mortality, 16-20% reduction in premature mortality, and 10-15% reduction in cardiovascular mortality, even in the presence of cardiometabolic risk factors. The joint associations, combining absence of the cardiometabolic risk factor and exercise, showed the greatest risk reduction (HRs ranging from 0.20 to 0.43).

**Conclusions:**

Regular exercise is consistently associated with reduced risk of all-cause, premature, and cardiovascular mortality, regardless of the presence of major cardiometabolic risk factors. These findings highlight exercise as a powerful and broadly applicable strategy for reducing mortality risk and support its integration into clinical and public health interventions, particularly in populations with high cardiometabolic burden.

## 1 Introduction

Cardiovascular diseases (CVDs) are the leading cause of mortality worldwide, accounting for over 20 million deaths during 2021 (1). The majority of these CVD-related deaths occur in low and middle-income countries (1). In Mexico, CVD is also the leading cause of mortality (2). In contrast to other North American countries, Mexico has experienced an increase in CVD-related mortality over the past 30 years (3). CVDs and their risk factors impose substantial economic costs; in Mexico, expenditures related to myocardial infarction, hypertension, and atrial fibrillation have been estimated at 2.5 billion USD, 1.7 billion USD, and 532 million USD, respectively (4). Additionally, CVDs are a major cause of premature mortality and disability in this country (5).

The development of CVD and mortality from cardiovascular events are associated with several risk factors (6). Some of these factors include chronic conditions that predispose individuals to both the development of CVD and increased mortality risk (7, 8). For instance, individuals with diabetes have a 1.48-fold higher risk of all-cause mortality and a 1.34-fold increased risk of cardiovascular events compared with those without diabetes (9). Sex-specific analyses indicate that women with diabetes have a 2.33-fold higher risk of all-cause mortality compared with women without diabetes, whereas men with diabetes have a 1.91-fold higher risk (10). Regarding premature mortality, women with diabetes have a 1.98-fold higher risk, while men have a 1.60-fold higher risk compared with individuals without diabetes (11). Also, hypertension is associated with a higher incidence of CVD and increased mortality (12, 13). Individuals with stage 1 and stage 2 hypertension have a 1.36-fold and 2.28-fold higher risk, respectively, of cardiovascular events compared to normotensive individuals (12), and stage 2 and stage 3 hypertension is associated with a 1.31-fold higher risk of all-cause mortality and a 1.49-fold higher risk of cardiovascular mortality (13). In addition, individuals with hypertension, prehypertension, or elevated-normal blood pressure have a significantly increased risk of premature cardiovascular death (14) and premature all-cause mortality (15).

Lifestyle factors are closely associated with health outcomes, unhealthy behaviors are strongly linked to mortality and cardiovascular health (16, 17). Tobacco use is a clear example; current smokers have a 2.07-fold higher risk of cardiovascular mortality compared with non-smokers (18). A cohort study in a Chinese population showed that smokers had an increased risk of premature mortality compared with non-smokers (19). Additionally, the number of cigarettes smoked per day is positively associated with both all-cause and CVD mortality (20). Obesity is another important risk factor associated with increased mortality (21). Several anthropometric indicators reflecting adiposity, including waist-to-hip ratio, body mass index (BMI), waist circumference, body roundness index, weight-adjusted waist index, relative fat mass, and body shape index, are associated with all-cause and CVD mortality (22). Adults with class II and III obesity have a higher risk of all-cause mortality compared to individuals with normal weight (23). Similarly, obesity is associated with an increased risk of CVD mortality, which rises progressively with higher BMI levels: 1.29-fold for BMI 30-34.9, 1.87-fold for BMI 35-39.9, and 2.21-fold for BMI ≥40 (24).

Exercising is also a well-established protective factor against all-cause, premature, and cardiovascular mortality. Compared with inactive individuals, those engaging in physical activity and exercise have a 33% lower risk of all-cause mortality and a 35% lower risk of CVD mortality (25). Regarding premature death, a longitudinal study found that active middle-aged individuals lived 2.1 years longer than those who were not (26). A dose-response relationship has also been established, with light and moderate-to-vigorous activity associated with 40% and 56% reduction in mortality risk, respectively (27). Despite its impact on cardiometabolic risk and mortality, exercise is often left out of risk assessment tools.

Although previous studies, including large-scale analyses from the Mexico City Prospective Study (MCPS), have established the protective effects of exercise on mortality, evidence regarding its role in the presence of established cardiometabolic risk factors remains limited in this high-risk population (28). Thus, the present study aims to explore the joint associations between exercise and classical cardiometabolic risk factors, including diabetes, hypertension, obesity, and smoking, with all-cause, premature, and cardiovascular mortality.

## 2 Methods

This study followed the Strengthening the Reporting of Observational Studies in Epidemiology (STROBE) guidelines.

### 2.1 Study design and procedures

This investigation is based on data from the MCPS, a large, population-based cohort of adults from the districts of Coyoacán and Iztapalapa, Mexico City. Trained nurses collected baseline data during household visits between 1998 and 2004. Vital status was tracked through December 31, 2020. Detailed information about the MCPS has been published elsewhere (28). Ethical approval was obtained from the Mexican Ministry of Health, the Mexican National Council of Science and Technology (approval number 0595 P-M), and the Central Oxford Research Ethics Committee (C99.260). All participants provided written informed consent at baseline.

### 2.2 Participants

A total of 159517 individuals aged 35 years or older were recruited. For this analysis, participants with missing information on exercise (n = 28), obesity (n = 2129), smoking (n = 140), education (n = 84), or vital status (n = 2070) were excluded. All other variables had complete information for all participants. A flow-chart of participant’s exclusion process is available in **Figure 1**. The final analytic sample comprised 155156 participants (67.1% women; mean age, 52.3 years).

**Figure&nbsp;1:**
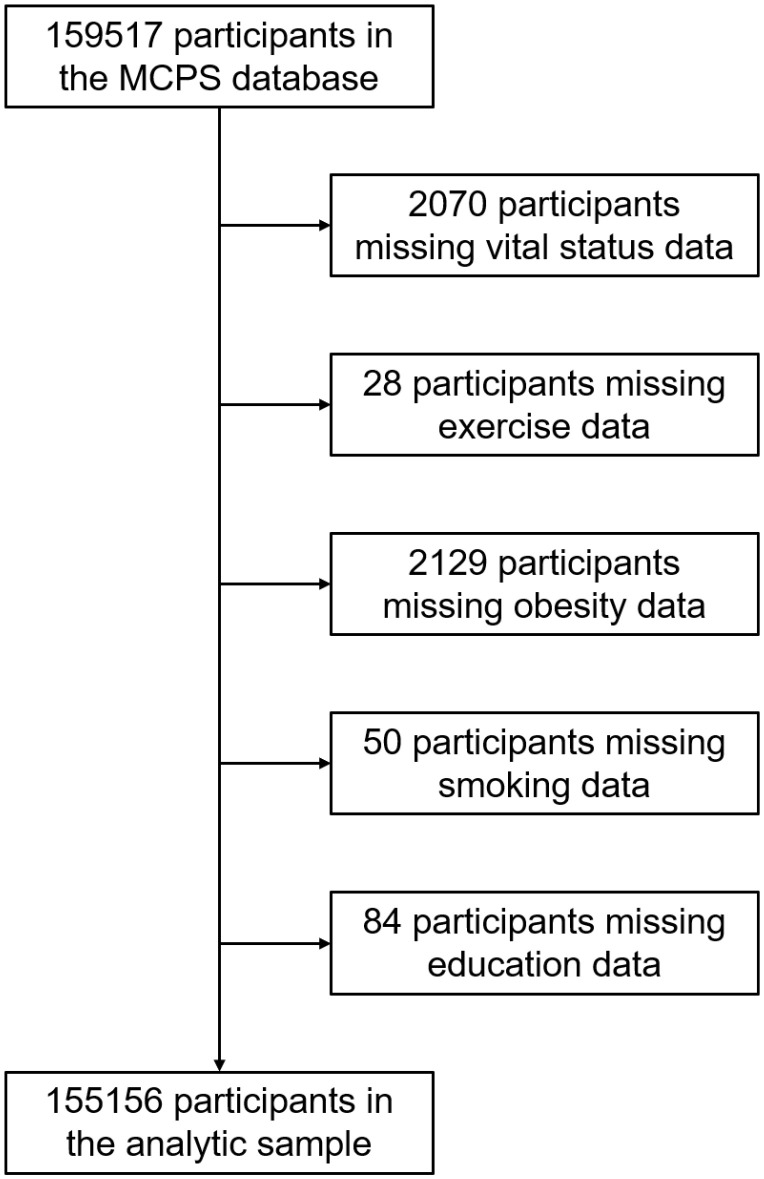
Participant flow-chart.

### 2.3 Measures

#### 2.3.1 Exercise and classical cardiometabolic modifiable risk factors (exposures)

Exercise behavior was assessed by self-report at baseline. Participants were first asked whether they engaged in exercise or sports (yes/no). Those who responded affirmatively were further asked about frequency, with response options of less than once per week, once or twice per week, or three or more times per week. For the main analysis, participants reporting less than one session per week were classified as non-exercisers, while those reporting at least one session per week were classified as exercisers. This categorization was used to maximize comparability with recent large epidemiological studies and to ensure sufficient statistical power across exposure groups (29–31).

We acknowledge that this measure captures only a limited dimension of exercise behavior and does not account for duration, intensity, type, total volume, or changes over follow-up. Therefore, it should be interpreted as a proxy of self-reported exercise frequency rather than total exercise behavior.

Classical cardiometabolic modifiable risk factors included previous diagnosis of hypertension (yes/no) and diabetes (yes/no), obesity assessed using the BMI (≥30 kg/m^2^), and smoking behavior (smoker/non-smoker). Joint categories of exposition were created by combining exercise behavior with each of the classical cardiometabolic modifiable risk factor (e.g., hypertension + non-exerciser, hypertension + exerciser, no hypertension + non-exerciser, no hypertension + exerciser).

#### 2.3.2 All- cause, premature and CVD mortality (outcomes)

Mortality outcomes in the MCPS were identified through record linkage with the national death registry of Mexico using name, sex and age for matching, which provides nearly complete coverage and medically certified causes of death (28). Follow-up extended from the date of the baseline survey to December 31, 2020. Deaths occurring during the follow-up period, including those during the COVID-19 pandemic (2020), were included in the analyses. COVID-19-related deaths were classified according to ICD-10 coding in the national death registry and contributed to all-cause mortality estimates. Three mortality endpoints were considered: all-cause mortality (death from any recorded cause), premature mortality (death occurring before the age of 75 years), and CVD mortality (ICD-10 codes I00-I99). Premature mortality was defined as death occurring before 75 years of age, a threshold commonly used in epidemiological and public health research to identify potentially avoidable or early deaths. Because the MCPS recruited adults aged 35–74 years, no participants were aged 75 years or older at baseline. For analyses of premature mortality, deaths occurring before age 75 were considered events, whereas participants who survived beyond age 75 were censored at their 75th birthday. Participants who did not experience the event were censored at the end of follow-up (December 31, 2020). Cox proportional hazards models used time since enrolment as the underlying time scale, with baseline age included as a covariate.

#### 2.3.3 Covariates

Sociodemographic covariates included age, sex, education, and household income. Age and sex were recorded by trained nurses at baseline. Education was self-reported at baseline and categorized into three groups: low (less than elementary school), medium (up to high school), and high (higher education). Household income was collected at baseline as weekly earnings in pesos.

### 2.4 Data analysis

Baseline characteristics were summarized as means (standard deviation) for continuous variables and as frequencies (percentages) for categorical variables. Person-years of follow-up were accrued from the date of the baseline survey to the date of death or December 31, 2020, whichever occurred first. For each cardiometabolic risk factor, a four-level joint exposure variable was created by combining risk factor status and exercise status. The category combining presence of the risk factor and no exercise was used as the reference group. These groups were designed to estimate joint exposure contrasts rather than to formally test multiplicative or additive interaction.

Cox proportional hazards regression models were used to estimate the hazard ratio (HR) and 95% confidence interval (95% CI) for all-cause, premature and CVD mortality, and primarily adjusted for sociodemographic covariates, including age (continuous), sex (categorical), education (categorical), and household income (tertiles). Findings are presented as HR or percentage of risks reduction calculated from HR (1-HR*100). To comprehensively assess independent associations within specific risk strata, multivariable models were further mutually adjusted for other classical cardiometabolic risk factors (i.e., when assessing the joint association of behavior and diabetes, models were additionally adjusted for hypertension, obesity, and smoking status). For CVD mortality, cause-specific Cox proportional hazards models were fitted. Cardiovascular deaths were treated as events, and deaths from non-cardiovascular causes were censored at the date of death. Accordingly, hazard ratios for cardiovascular mortality should be interpreted as cause-specific hazard ratios rather than as direct estimates of absolute risk or cumulative incidence.

Model assumptions were assessed using graphical diagnostic procedures. For categorical covariates, log-minus-log survival plots were visually inspected to evaluate whether hazards remained approximately proportional over follow-up. For continuous covariates, partial Schoenfeld residuals were plotted against survival time and inspected for systematic departures from a horizontal trend. No relevant violation of the proportional hazards assumption was detected. The linearity assumption for continuous covariates was assessed by plotting Martingale residuals against age, with no meaningful departure from linearity observed. Sensitivity analyses were conducted by excluding deaths occurring within the first 2 and 5 years of follow-up to reduce potential reverse causation. All statistical analyses were performed in August 2025 using SPSS software, version 29.0 (IBM Corp.). A two-sided p < 0.05 was considered statistically significant.

## 3 Results

Participants’ characteristics are presented in **Table 1**. At baseline, 77.7% were classified as non-exercisers, while 22.3% reported exercising at least once per week. The prevalence of classical cardiometabolic risk factors was 21.0% with hypertension, 13.4% with diabetes, 36.7% with obesity, and 31.3% were current smokers. During a median follow-up of 18.26 years (interquartile range: 17.49, 19.57), there were 27510 deaths, of which 13970 were premature (<75 years) and 9192 were attributed to cardiovascular causes.

**Table 1:** Baseline characteristics of the participants.

Variable	Mean ± SD or n (%)
Age	52.3 ± 13.1
Sex	
Female	104170 (67.1)
Male	50986 (32.9)
Income (pesos/week)	1686.7 ± 3722.0
Education	
Low	51402 (33.1)
Medium	79687 (51.4)
High	24022 (15.5)
Hypertension	
No	122501 (79.0)
Yes	32655 (21.0)
Diabetes	
No	134403 (86.6)
Yes	20753 (13.4)
Obesity	
No	98139 (63.3)
Yes	57017 (36.7)
Smoker	
No	106623 (68.7)
Yes	48533 (31.3)
Physical activity frequency	
< 1/week	120534 (77.7)
1+/week	34622 (22.3)

SD, standard deviation.

Exercisers had significantly lower mortality than non-exercisers (**Table 2**). In adjusted models, the reduction in all-cause mortality among exercisers ranged from 12% to 16% (HRs 0.84-0.88) even in the presence of cardiometabolic risk factors. For premature mortality, the corresponding reduction was 16% to 20% (HRs 0.80-0.84), while for CVD mortality it was 10% to 15% (HRs 0.85-0.90). These estimates were consistent in direction and magnitude across all four risk factors. The lowest hazard ratios were generally observed for the joint category combining absence of the cardiometabolic risk factor and self-reported exercise at least once per week, with HRs ranging from 0.20 to 0.43 depending on the outcome.

**Table 2:** Joint associations between exercising and classical cardiometabolic risk factors with all-cause, premature and cardiovascular mortality.

Exposure group	n	All-cause mortality			Premature mortality			Cardiovascular mortality		
		Deaths	Unadjusted HR (95% CI)	Adjusted HR (95% CI)	Deaths	Unadjusted HR (95% CI)	Adjusted HR (95% CI)	Deaths	Unadjusted HR (95% CI)	Adjusted HR (95% CI)
Hypertension + Non-exerciser	25405	7712	1.00 (ref.)	1.00 (ref.)	3337	1.00 (ref.)	1.00 (ref.)	3051	1.00 (ref.)	1.00 (ref.)
Hypertension + Exerciser	7250	1994	0.88 (0.84-0.92)	0.85 (0.81-0.89)	798	0.81 (0.75-0.88)	0.81 (0.75-0.88)	821	0.92 (0.85-0.99)	0.88 (0.81-0.95)
No hypertension + Non-exerciser	95129	14337	0.45 (0.43-0.46)	0.78 (0.76-0.80)	7981	0.58 (0.55-0.60)	0.69 (0.66-0.72)	4227	0.33 (0.32-0.35)	0.62 (0.59-0.66)
No hypertension + Exerciser	27372	3467	0.37 (0.35-0.38)	0.66 (0.64-0.69)	1854	0.46 (0.43-0.48)	0.56 (0.52-0.59)	1093	0.29 (0.27-0.31)	0.56 (0.53-0.61)
Diabetes + Non-exerciser	16481	6914	1.00 (ref.)	1.00 (ref.)	4113	1.00 (ref.)	1.00 (ref.)	2170	1.00 (ref.)	1.00 (ref.)
Diabetes + Exerciser	4272	1688	0.91 (0.86-0.96)	0.88 (0.83-0.92)	945	0.86 (0.80-0.92)	0.84 (0.78-0.90)	552	0.95 (0.86-1.04)	0.90 (0.82-0.99)
No diabetes + Non-exerciser	104053	15135	0.28 (0.27-0.29)	0.42 (0.41-0.44)	7205	0.23 (0.22-0.24)	0.25 (0.24-0.26)	5108	0.30 (0.29-0.32)	0.49 (0.46-0.51)
No diabetes + Exerciser	30350	3773	0.24 (0.23-0.25)	0.36 (0.34-0.37)	1707	0.18 (0.17-0.19)	0.20 (0.19-0.21)	1362	0.27 (0.25-0.29)	0.43 (0.40-0.46)
Obesity + Non-exerciser	47030	8252	1.00 (ref.)	1.00 (ref.)	4723	1.00 (ref.)	1.00 (ref.)	2705	1.00 (ref.)	1.00 (ref.)
Obesity + Exerciser	9987	1671	0.94 (0.89-0.99)	0.86 (0.81-0.90)	885	0.87 (0.81-0.94)	0.82 (0.76-0.88)	588	1.01 (0.92-1.11)	0.90 (0.82-0.98)
No obesity + Non-exerciser	73504	13797	1.08 (1.05-1.11)	0.91 (0.88-0.93)	6595	0.90 (0.87-0.94)	0.88 (0.84-0.91)	4573	1.09 (1.04-1.15)	0.88 (0.84-0.93)
No obesity + Exerciser	24635	3790	0.86 (0.82-0.89)	0.77 (0.74-0.80)	1767	0.70 (0.66-0.74)	0.70 (0.67-0.75)	1326	0.92 (0.86-0.98)	0.79 (0.74-0.85)
Smoker + Non-exerciser	36976	5789	1.00 (ref.)	1.00 (ref.)	3854	1.00 (ref.)	1.00 (ref.)	1738	1.00 (ref.)	1.00 (ref.)
Smoker + Exerciser	11557	1461	0.79 (0.74-0.83)	0.84 (0.79-0.89)	947	0.77 (0.71-0.82)	0.80 (0.75-0.86)	450	0.81 (0.73-0.89)	0.85 (0.77-0.94)
Non-smoker + Non-exerciser	83558	16260	1.28 (1.25-1.32)	0.92 (0.89-0.95)	7464	0.88 (0.85-0.92)	0.91 (0.88-0.95)	5540	1.46 (1.38-1.54)	0.90 (0.85-0.95)
Non-smoker + Exerciser	23065	4000	1.12 (1.07-1.16)	0.78 (0.75-0.82)	1705	0.72 (0.68-0.76)	0.74 (0.69-0.78)	1464	1.36 (1.27-1.46)	0.81 (0.76-0.88)

CI, confidence interval; HR, hazard ratio.

Analysis was adjusted for sex, age, education, income and cardiometabolic risk factors.

### 3.1 Exercise and hypertension

Compared with individuals with hypertension who did not exercise, those with hypertension who exercised had lower risks of all-cause mortality (HR 0.85, 95% CI 0.81-0.89), premature mortality (HR 0.81, 95% CI 0.75-0.88), and CVD mortality (HR 0.88, 95% CI 0.81-0.95). The combination of exercise and not having hypertension was also associated with lower mortality across all outcomes (all-cause: HR 0.66, 95% CI 0.64-0.69; premature: HR 0.56, 95% CI 0.52-0.59; CVD: HR 0.56, 95% CI 0.53-0.61).

### 3.2 Exercise and diabetes

Exercising participants with diabetes had reduced risks of all-cause (HR 0.88, 95% CI 0.83-0.92) and premature mortality (HR 0.84, 95% CI 0.78-0.90), compared with non-exercisers. CVD mortality was also modestly lower (HR 0.90, 95% CI 0.82-0.99). Compared with participants with diabetes who did not exercise, participants without diabetes who reported exercising at least once per week had lower hazards across all mortality outcomes (all-cause: HR 0.36, 95% CI 0.34-0.37; premature: HR 0.20, 95% CI 0.19-0.21; CVD: HR 0.43, 95% CI 0.40–0.46).

### 3.3 Exercise and obesity

In obese participants, exercise was associated with lower all-cause (HR 0.86, 95% CI 0.81-0.90) and premature mortality (HR 0.82, 95% CI 0.76-0.88). A weaker but still protective association was observed for CVD mortality (HR 0.90, 95% CI 0.82-0.98). Compared with participants with obesity who did not exercise, participants without obesity who reported exercising at least once per week had lower risks of all-cause (HR 0.77, 95% CI 0.74-0.80), premature (HR 0.70, 95% CI 0.67-0.75), and CVD mortality (HR 0.79, 95% CI 0.74-0.85).

### 3.4 Exercise and smoking

Exercising smokers experienced significantly lower mortality compared with non-exercising smokers (all-cause: HR 0.84, 95% CI 0.79-0.89; premature: HR 0.80, 95% CI 0.75-0.86; CVD: HR 0.85, 95% CI 0.77-0.94). Compared with smokers who did not exercise, non-smokers who reported exercising at least once per week had lower risks of all-cause (HR 0.78, 95% CI 0.75-0.82), premature (HR 0.74, 95% CI 0.69-0.78), and CVD mortality (HR 0.81, 95% CI 0.76-0.88).

### 3.5 Sensitivity analyses

Sensitivity analyses excluding deaths within the first 2 and 5 years of follow-up yielded results similar to the main analysis (**Table 3**). Effect estimates were stable in terms of direction, magnitude, and statistical significance, reducing concerns of reverse causality.

**Table 3:** 2-year and 5-year reverse causality sensitivity analysis for the joint associations between exercising and classical cardiometabolic risk factors with all-cause, premature and cardiovascular mortality.

Exposure group	All-cause mortality		Premature mortality		Cardiovascular mortality	
	2-year RC* HR (95% CI)	5-year RC^†^ HR (95% CI)	2-year RC* HR (95% CI)	5-year RC^†^ HR (95% CI)	2-year RC* HR (95% CI)	5-year RC^†^ HR (95% CI)
Hypertension + Non-exerciser	1.00 (ref.)	1.00 (ref.)	1.00 (ref.)	1.00 (ref.)	1.00 (ref.)	1.00 (ref.)
Hypertension + Exerciser	0.88 (0.84-0.93)	0.89 (0.84-0.94)	0.84 (0.78-0.92)	0.85 (0.77-0.93)	0.90 (0.83-0.98)	0.90 (0.82-0.98)
No hypertension + Non-exerciser	0.80 (0.77-0.82)	0.82 (0.79-0.85)	0.72 (0.69-0.75)	0.76 (0.72-0.80)	0.64 (0.61-0.67)	0.66 (0.62-0.70)
No hypertension + Exerciser	0.69 (0.66-0.72)	0.72 (0.69-0.76)	0.59 (0.56-0.63)	0.63 (0.58-0.67)	0.59 (0.55-0.63)	0.61 (0.56-0.66)
Diabetes + Non-exerciser	1.00 (ref.)	1.00 (ref.)	1.00 (ref.)	1.00 (ref.)	1.00 (ref.)	1.00 (ref.)
Diabetes + Exerciser	0.91 (0.86-0.96)	0.93 (0.88-0.99)	0.88 (0.81-0.94)	0.89 (0.82-0.97)	0.92 (0.83-1.01)	0.93 (0.83-1.03)
No diabetes + Non-exerciser	0.43 (0.42-0.44)	0.44 (0.42-0.45)	0.25 (0.24-0.26)	0.25 (0.24-0.26)	0.49 (0.46-0.52)	0.51 (0.48-0.54)
No diabetes + Exerciser	0.37 (0.35-0.39)	0.38 (0.36-0.40)	0.20 (0.19-0.22)	0.20 (0.19-0.21)	0.45 (0.42-0.48)	0.46 (0.43-0.50)
Obesity + Non-exerciser	1.00 (ref.)	1.00 (ref.)	1.00 (ref.)	1.00 (ref.)	1.00 (ref.)	1.00 (ref.)
Obesity + Exerciser	0.88 (0.83-0.93)	0.88 (0.84-0.94)	0.84 (0.78-0.91)	0.84 (0.78-0.91)	0.91 (0.83-0.99)	0.90 (0.82-0.99)
No obesity + Non-exerciser	0.88 (0.85-0.90)	0.83 (0.81-0.86)	0.83 (0.80-0.87)	0.79 (0.75-0.81)	0.87 (0.82-0.91)	0.84 (0.79-0.89)
No obesity + Exerciser	0.77 (0.74-0.80)	0.74 (0.71-0.77)	0.69 (0.65-0.73)	0.64 (0.60-0.69)	0.80 (0.74-0.86)	0.77 (0.71-0.83)
Smoker + Non-exerciser	1.00 (ref.)	1.00 (ref.)	1.00 (ref.)	1.00 (ref.)	1.00 (ref.)	1.00 (ref.)
Smoker + Exerciser	0.86 (0.81-0.91)	0.87 (0.82-0.93)	0.83 (0.77-0.89)	0.84 (0.79-0.91)	0.86 (0.78-0.96)	0.87 (0.77-0.97)
Non-smoker + Non-exerciser	0.91 (0.88-0.95)	0.91 (0.88-0.94)	0.92 (0.88-0.96)	0.91 (0.87-0.96)	0.90 (0.85-0.96)	0.90 (0.84-0.96)
Non-smoker + Exerciser	0.81 (0.77-0.84)	0.81 (0.77-0.85)	0.76 (0.72-0.81)	0.75 (0.70-0.80)	0.84 (0.78-0.90)	0.83 (0.77-0.90)

CI, confidence interval; HR, hazard ratio; RC, reverse causality.

Analysis was adjusted for sex, age, education, income and cardiometabolic risk factors.

*n=152934.

^†^ n=148911.

## 4 Discussion

This large prospective cohort study of more than 155, 000 Mexican adults, followed for over 18 years, provides robust evidence that self-reported exercise at least once per week was associated with a significantly lower risk of all-cause, premature, and cardiovascular disease (CVD) mortality. A notable finding was the consistency of these associations across the four classical cardiometabolic risk factors examined (hypertension, diabetes, obesity, and smoking), with exercisers experiencing approximately 10–20% lower mortality risk than non-exercisers regardless of baseline risk status. Furthermore, were associated with substantial differences in mortality risk. These findings suggest that the observed associations are broadly applicable and largely independent of underlying cardiometabolic risk profiles. The observed associations are biologically plausible, as self-reported exercise at least once per week is associated with improvements in vascular function, cardiorespiratory fitness, metabolic regulation, and systemic inflammation, thereby reducing susceptibility to fatal health events (32–36).

Beyond its effects on individual cardiometabolic risk factors, regular exercise exerts a wide range of systemic adaptations that may contribute to the lower mortality risk observed in this study. Exercise is associated with improved cardiorespiratory fitness, one of the strongest predictors of longevity, by improving cardiac output, oxygen delivery, mitochondrial function, and skeletal muscle efficiency (37). It also promotes favorable metabolic adaptations, including improved glucose uptake, insulin sensitivity, lipid metabolism, and body composition, which collectively are associated with a lower burden of metabolic disease (38). At the vascular level, exercise is associated with improved endothelial function, increased arterial compliance, and better blood pressure regulation, thereby potentially contributing to slower progression of atherosclerotic disease and cardiovascular events (39). Furthermore, regular physical activity is associated with modulation of chronic low-grade inflammation, oxidative stress, autonomic nervous system balance, and immune function, all of which have been implicated in the development of cardiovascular disease, cancer, and other major causes of premature death (40). These multisystem effects may help explain why exercise is consistently associated with lower mortality risk across populations with diverse health profiles and risk factor burdens.

When analyzed within each risk factor category, the protective associations demonstrated a clear and coherent pattern. In the hypertensive cohort, physical exertion was found to be associated with a 15% decrease in all-cause mortality, a 19% decrease in premature mortality, and a 12% decrease in cardiovascular mortality. A similar pattern was observed among participants with diabetes, for whom self-reported exercise at least once per week was associated with a reduction in all-cause mortality of 12%, premature mortality of 16%, and cardiovascular mortality of almost 10%. These findings are consistent with previous evidence suggesting that self-reported exercise at least once per week is associated with lower risk of negative outcomes related to hypertension and diabetes (41, 42). Furthermore, these findings extend existing knowledge by demonstrating that such benefits are also evident in a Latin American population with a high cardiometabolic burden and increasing CVD mortality trends (43, 44). The observed benefits in these groups may reflect exercise-induced reductions in arterial stiffness (45), improvements in insulin sensitivity and glycemic control (46), and attenuation of endothelial dysfunction (47), which are key drivers of CVD mortality in hypertensive and diabetic populations.

Among participants with obesity, exercise was associated with a 14% reduction in all-cause mortality and an 18% reduction in premature mortality, as well as more modest yet still significant 10% reduction in CVD mortality. This pattern is particularly relevant in populations with a high prevalence of obesity and supports the concept that self-reported exercise at least once per week may partially attenuate the increased mortality risk associated with excess adiposity (48). In a similar manner, among smokers, individuals who self-reported exercise at least once per week demonstrated a 16% reduction in all-cause mortality, a 20% reduction in premature mortality, and a 15% reduction in CVD mortality when compared with smokers who did not exercise. These findings serve to reinforce the notion that physical activity is associated with measurable differences in mortality risk, even in the presence of adverse behavioral factors such as smoking (49). These patterns may be explained by the ability of exercise to improve cardiorespiratory capacity (50), reduce visceral adiposity (50), enhance autonomic balance, and counteract chronic inflammation and oxidative stress (51), thereby increasing physiological resilience even when adverse risk exposures persist.

The joint exposure analyses also showed that the lowest mortality hazards were generally observed among participants who both did not have the cardiometabolic risk factor and reported exercising at least once per week, compared with the corresponding high-risk reference group. The HR for these groups approached reductions of 30-60%, depending on the outcome. While this may partly reflect lower baseline risk, it also suggests that self-reported exercise at least once per week may be associated with greater relative differences in risk when present in already favorable cardiometabolic profiles (37), amplifying survival benefits (52). The robust correlations observed for premature mortality are of particular pertinence, as they underscore the potential of self-reported exercise at least once per week as a pragmatic approach to augmenting healthy life expectancy and postponing premature mortality, a major public health concern in Mexico (28, 53).

The consistency of findings across all mortality outcomes, the reproducibility across independent risk factor strata, and the stability of estimates in sensitivity analyses excluding early deaths collectively serve to reinforce the plausibility of an association between self-reported exercise at least once per week and lower mortality risk. These results, consistent with previous research (32, 54, 55), support exercise as a universally beneficial behavior, associated with lower mortality risk regardless of underlying cardiometabolic risk status, while showing particularly strong associations in populations facing high cardiometabolic vulnerability. Together, the biological, behavioral, and clinical coherence of these results supports exercise as a health behavior consistently associated with lower mortality risk across diverse cardiometabolic risk profiles (56).

### 4.1 Practical implications

The consistent reductions in all-cause, premature, and CVD mortality across individuals with hypertension, diabetes, obesity, and smoking suggest exercise as a potential core therapeutic and preventive strategy in cardiometabolic risk management. These findings underscore the notion that exercise should not be regarded exclusively as a complementary lifestyle recommendation; rather, it should be conceptualized as a clinically relevant intervention with quantifiable mortality benefits. In practical terms, this supports the integration of physical activity counselling and exercise prescription into routine clinical care, alongside pharmacological treatment and traditional risk factor control.

From a broader public health perspective, the substantial benefits observed, especially regarding premature mortality, highlight the potential impact of promoting exercise as a population-level strategy to reduce CVD burden in Mexico. This warrants the reinforcement of policies and programs that promote active lifestyles, such as increasing access to safe environments for active commuting and physical activity (e.g., cycling paths, outdoor parks), integrating exercise within primary healthcare services (e.g., referral schemes), and enabling community-based initiatives that deliver and encourage participation in exercise (e.g., community exercise programs).

### 4.2 Strengths, limitations and future lines of research

The present study has several strengths. MCPS is a population-based cohort comprising over 155, 000 participants, with an extensive follow-up period exceeding 18 years. This allows for the evaluation of long-term associations between self-reported exercise at least once per week and mortality, with considerable statistical power. Mortality outcomes were obtained through linkage with the national death registry, ensuring near-complete ascertainment and reliable classification of causes of death. Furthermore, the study examined the joint associations of self-reported exercise at least once per weekwith multiple major cardiometabolic risk factors and evaluated different mortality endpoints, providing a comprehensive and clinically relevant perspective. The consistency of results across subgroups and their stability in sensitivity analyses further reinforce the robustness of the findings.

Several limitations should be considered when interpreting these findings. First, the main exposure (exercise behaviour) was assessed by self-report at baseline using a simplified frequency-based measure, which is subject to misclassification and does not capture key dimensions of physical activity, including intensity, duration, type, total volume, or changes over time. This may have introduced non-differential measurement error and regression dilution bias, potentially attenuating the observed associations. Second, although BMI was derived from objectively measured height and weight obtained by trained nurses, several baseline cardiometabolic risk factors were self-reported and may be affected by reporting inaccuracies. Third, changes in exercise behaviour and risk factor status during follow-up were not captured, which may have influenced the observed associations. In addition, residual confounding cannot be fully excluded despite adjustment for major sociodemographic and cardiometabolic variables, as unmeasured lifestyle and health-related factors may have contributed to the associations observed. Finally, although the cohort is large and population-based, it was drawn from specific districts of Mexico City, which may limit generalisability to other populations and settings.

Residual confounding remains a key limitation of this observational study. Although we adjusted for several important covariates, including age, sex, education, income, and cardiometabolic risk factors, we cannot exclude the possibility that unmeasured or incompletely measured variables may have influenced the observed associations. In particular, baseline health status, undiagnosed disease, medication use, dietary patterns, alcohol consumption, occupational and transport-related physical activity, healthcare utilisation, and health-related behaviours may confound the relationship between physical activity and mortality. In addition, reverse causation cannot be fully ruled out, as individuals with poorer underlying health may be less likely to engage in physical activity, even though sensitivity analyses excluding early follow-up years were performed. Therefore, the findings should be interpreted as associative rather than causal.

These limitations, therefore, provide a framework for establishing future research priorities. Further research is required into the incorporation of objective measures of exercise and repeated assessments over time. Such studies would serve to refine exposure estimation and evaluate how changes in exercise behavior influence mortality risk. Subsequent research should investigate dose-response relationships and elucidate the differential impact of exercise types, frequency, and intensity. Finally, implementation and intervention studies in real-world healthcare and community settings are essential to determine the effectiveness, feasibility, and scalability of exercise-based strategies, particularly in middle-income countries experiencing a high cardiometabolic burden, such as Mexico.

## 5 Conclusion

This large prospective cohort study provides robust evidence that self-reported exercise at least once per week is associated with lower risks of all-cause, premature, and CVD mortality among Mexican adults, including those with major cardiometabolic risk factors such as hypertension, diabetes, obesity, and smoking. Findings emphasize the need to incorporate exercise as a fundamental component within both primary and secondary CVD prevention strategies and underscore its potential to contribute to reducing premature mortality and improving population health.

## Data Availability

The original contributions presented in the study are included in the article/Supplementary Material. Further inquiries can be directed to the corresponding author.
